# Clinical impact of diarrhea during enteral feeding after esophagectomy

**DOI:** 10.1007/s10147-023-02428-5

**Published:** 2023-11-23

**Authors:** Ryoma Haneda, Yoshihiro Hiramatsu, Sanshiro Kawata, Wataru Soneda, Eisuke Booka, Tomohiro Murakami, Tomohiro Matsumoto, Yoshifumi Morita, Hirotoshi Kikuchi, Hiroya Takeuchi

**Affiliations:** 1 Department of Surgery, Hamamatsu University School of Medicine, Hamamatsu, Shizuoka Japan; 2 Department of Perioperative Functioning Care and Support, Hamamatsu University School of Medicine, 1-20-1 Handayama, Higashi-Ku, Hamamatsu, Shizuoka 431-3192 Japan

**Keywords:** Esophagectomy, Enteral feeding, Diarrhea, Malnutrition, Survival

## Abstract

**Background:**

Enteral feeding (EF) is recommended to enhance nutritional status after esophagectomy; however, diarrhea is a common complication of EF. We investigated the clinical and prognostic impact of diarrhea during EF after esophagectomy.

**Methods:**

One hundred and fifty-two patients who underwent transthoracic esophagectomy were enrolled. The King's stool chart was used for stool characterization. The short- and long-term outcomes were compared between a non-diarrhea (Group N) and diarrhea group (Group D).

**Results:**

A higher dysphagia score (≥ 1) was observed more frequently in Group D than in Group N (45.7% vs. 19.8%, *p* = 0.002). Deterioration of serum total protein, serum albumin, serum cholinesterase, and the prognostic nutritional index after esophagectomy was greater in Group D than in Group N (*p* = 0.003, 0.004, 0.014, and 0.001, respectively). Patients in Group D had significantly worse overall survival (OS) and recurrence-free survival (RFS) than those in Group N (median survival time (MST): OS, 21.9 vs. 30.6 months, *p* = 0.001; RFS, 12.4 vs. 27.7 months, *p* < 0.001). In stratified analysis due to age, although there was no difference in OS with or without diarrhea in young patients (MST: 24.1 months in a diarrhea group vs. 33.6 months in a non-diarrhea group, *p* = 0.218), patients in a diarrhea group had significantly worse OS than those in a non-diarrhea group in elderly patients (MST: 17.8 months vs. 27.9 months, *p* < 0.001).

**Conclusions:**

Diarrhea during EF can put elderly patients at risk of postoperative malnutrition and a poor prognosis after esophagectomy.

**Supplementary Information:**

The online version contains supplementary material available at 10.1007/s10147-023-02428-5.

## Introduction

Global cancer statistics show that esophageal cancer is the sixth leading cause of cancer-related deaths worldwide [1]. Patients with esophageal cancer have many nutritional risks. Masses often cause solid food obstruction preoperatively, and oral intake must be ceased for a certain period postoperatively to protect the anastomosis site [2]. Additionally, transthoracic esophagectomy, recognized as a principal treatment for esophageal cancer [3, 4], is more invasive, resulting in a systemic inflammatory response and poses a risk for postoperative complications [5]. We previously reported that enhancing postoperative nutritional status in patients with preoperative malnutrition leads to a better prognosis after esophagectomy [6]. Other studies have suggested that nutritional management in the early postoperative period is important to enhance cell-mediated immunity after esophagectomy [5, 7, 8]. Enteral feeding (EF) has been recommended as supportive therapy for patients after surgery or in intensive care [2, 5, 7, 9]. EF has been shown to promote nitrogen retention, restore immune function, and accelerate wound healing [10]. Postoperative EF has also been shown to reduce the risk of septic complications after esophagectomy [2].

However, the complications associated with specific EF methods may diminish the intended benefits. Diarrhea is a major complication of EF [10, 11]. The incidence of diarrhea during EF in intensive care is reported to be 2–95% [2, 10–12]. In addition, diarrhea has been linked to malnutrition [2, 10]. In intensive care, the combination of stress and malnutrition is associated with a negative energy balance, which leads to delayed wound healing, prolonged hospital stay, and higher healthcare costs [12]. Furthermore, malnutrition is associated with increased morbidity and mortality during critical illness [2, 12]. However, the clinical significance of diarrhea during EF after esophagectomy remains unknown.

This study hypothesizes that diarrhea during EF after esophagectomy could be a risk factor for postoperative malnutrition and poor prognosis. Therefore, we investigated the associations between diarrhea during EF with short- and long-term outcomes.

## Patients and methods

### Patients

Between January 2017 and December 2021, 178 patients with esophageal and esophagogastric junction cancer (EGJC) were retrospectively reviewed. All patients underwent esophagogastroduodenoscopy (EGD) and computed tomography (CT) from the neck to the pelvis to determine the clinical stage. The clinical and pathological stages were determined based on the Union for International Cancer Control TNM classification of malignant tumors, 8th edition [13].

**Fig. 1:**
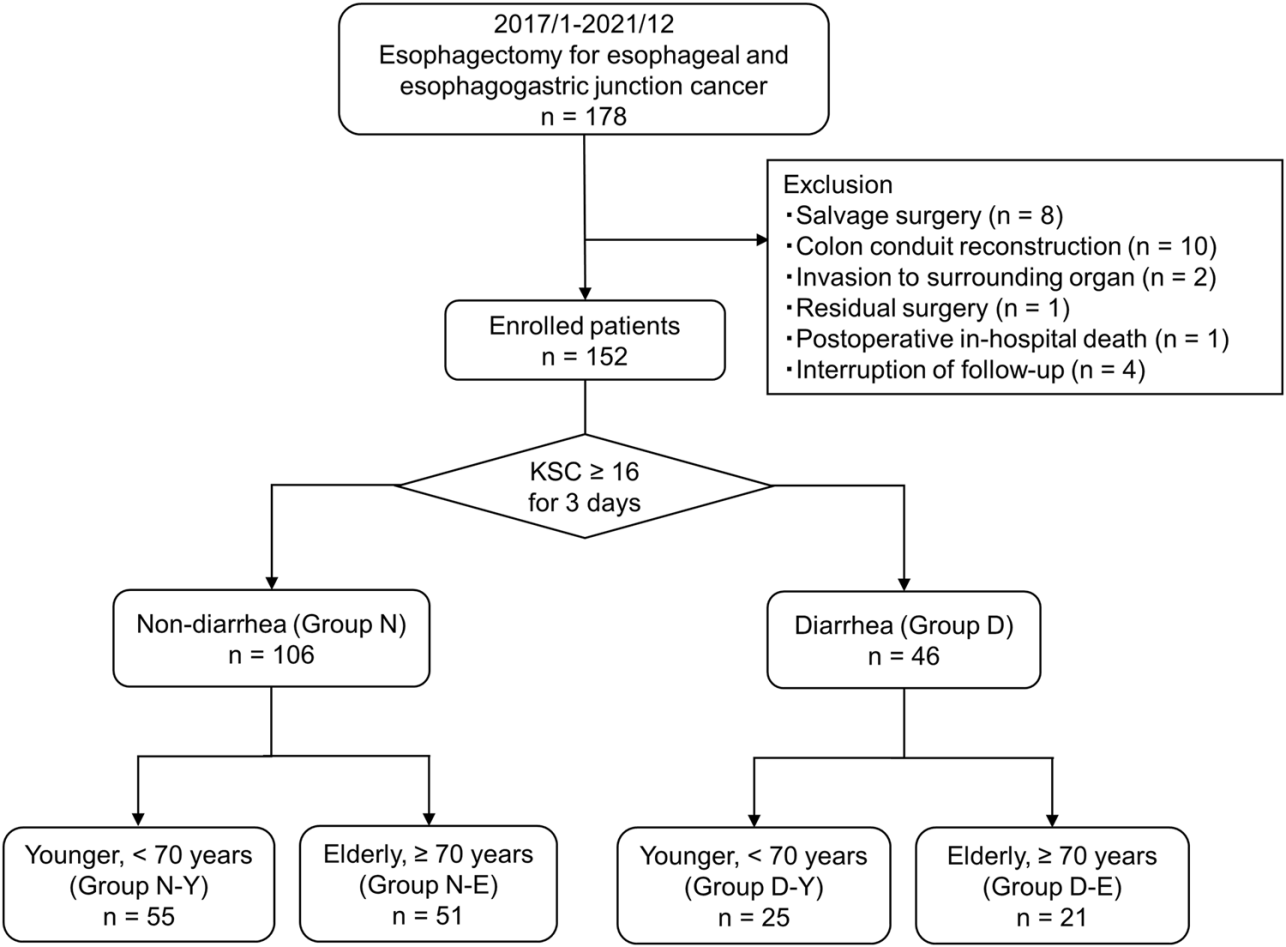
Study flow diagram

Patients who met the following criteria were enrolled in this study: (1) age > 20 years, (2) Eastern Cooperative Oncology Group performance status ≤ 1, (3) radical esophagectomy, (4) reconstruction via a gastric conduit, (5) no double cancer, (6) no prior irradiation, (7) one-stage surgery, and (8) follow-up for more than 1 year. Patients were excluded from the study based on the following criteria: salvage surgery (n = 8), colon conduit reconstruction (n = 10), invasion to surrounding organs (n = 2), residual disease (n = 1), postoperative in-hospital death (n = 1), and interruption of follow-up within 1 year (n = 4). Finally, 152 patients were included in this study (Fig. 1).

### Multidisciplinary treatment

In accordance with the esophageal cancer practice guidelines 2017 in Japan, neoadjuvant chemotherapy (NAC) was administered to patients with non-Stage I squamous cell carcinoma (SCC) [3, 4]. At our institution, patients with adenocarcinoma with bulky lymph node (LN) metastases underwent NAC. For SCC, the treatment regimens were a combination of cisplatin and 5-fluorouracil or a combination of docetaxel, cisplatin, and 5-fluorouracil for SCC and a combination of S-1 and oxaliplatin for adenocarcinoma. A right transthoracic subtotal esophagectomy with 2- or 3-field LNs dissection was performed as a standard surgical procedure at our institution [14, 15]. Upper, middle, and lower mediastinal LNs and abdominal LNs were routinely dissected. The upper mediastinal region included the upper thoracic paraesophageal nodes, and left and right paratracheal nodes; the middle mediastinal region included the middle thoracic paraesophageal nodes, subcarinal nodes, and main bronchus nodes; and the lower mediastinal region included the lower thoracic paraesophageal nodes, posterior mediastinal nodes, and supradiaphragmatic nodes. In the abdominal region, bilateral paracardial nodes, lesser curvature nodes, and LNs along the left gastric artery, common hepatic artery, celiac artery, and proximal splenic artery were dissected. Except for patients with low surgical tolerance or high surgical risk, bilateral cervical LNs dissection was generally performed for advanced cancer or superficial cancer in the middle or upper thoracic esophagus. Gastric conduit reconstruction via the posterior mediastinal route was performed with hand-sewn anastomosis in the neck. The retrosternal route was selected when the risk of anastomotic leakage (AL) was considered high, such as in those who took steroids or suspected insufficient blood flow in the gastric conduit. In posterior mediastinal route reconstruction, a 12-Fr jejunostomy catheter was inserted into the proximal jejunum. Further, we inserted this into the gastric antrum in retrosternal route reconstruction. Following esophagectomy, cefazolin 1 g was administered twice daily via a peripheral intravenous line for 3 days as a prophylactic antibiotic.

The Clavien–Dindo classification was used to assess postoperative complications such as pneumonia, AL, and surgical site infection (SSI). Further, postoperative complications of grade ≥ II were identified [16]. Postoperative body weight (BW), body mass index (BMI), serum total protein, serum albumin, serum cholinesterase, prognostic nutritional index (PNI) were used to assess nutritional status [17]. Additionally, the serum C-reactive protein (CRP) and neutrophil-to-lymphocyte ratio (NLR) were used as inflammatory markers. BW and BMI were measured before and at 1 and 3 months after surgery. Other parameters were measured in blood samples taken before and 1 month after surgery. These parameters were measured after NAC but before surgery in patients who received NAC. Patients were divided into high and low groups using median values for PNI and NLR and the institutional reference level for CRP (0.3 mg/dl).

### Perioperative nutritional support

In April 2017, a multidisciplinary support team was established to prevent complications and improve nutritional status [18]. Before treatment, dietitians assessed the oral intake and recommended oral nutritional supplements for those whose calorie intake was insufficient. Patients with severe dysphagia caused by massive tumors were admitted early, and EF via a nasogastric tube was performed during NAC. Other patients were admitted 5 days prior to surgery and received oral nutritional support the following day. An elemental diet was started at 10 ml/h (240 kcal/day) on the day of surgery via a jejunostomy tube. EF speed was gradually increased to 50 ml/h (1200 kcal/day) based on the patient’s abdominal condition (Online Resource 1). EF was reduced or stopped temporarily because of diarrhea, abdominal fullness, chylothorax, or anorexia. On postoperative day 7, the EF agent was gradually changed from an elemental diet to a fat-containing agent. CT and upper gastrointestinal contrast imaging were performed on postoperative day 7 to ensure that there are no complications such as AL. Video-fluoroscopic and video-endoscopic examinations of the swallowing function were performed before starting oral intake (Online Resource 1). However, patients with AL were deferred from starting the oral diet. When diarrhea occurred, the EF speed was reduced first. We changed the EF agent or started probiotics if no improvement was observed. A multidisciplinary team conference was held to discuss the required calories, nutritional status, dietary intake, EF agents, and stool condition of the patients [6, 18]. EF was continued in patients after hospital discharge until oral intake was satisfactory. The degree of preoperative food passage obstruction was assessed using the dysphagia score (Table 1).

**Table 1:** Dysphagia score

Score	Degree of food obstruction
0	Able to eat normal diet
1	Able to swallow some solid foods
2	Able to swallow only semi solid foods
3	Able to swallow liquids only
4	Unable to swallow anything

### Assessment of fecal output

The King’s Stool Chart (KSC), which incorporates the frequency, consistency, and weight of fecal output during EF, was used to assess diarrhea (Table 2) [19]. The daily fecal score was calculated by scoring fecal conditions into 12 categories and summing the daily scores. Patients with KSC scored ≥ 16 should have their EF speed, or agents changed [19, 20]. In this study, diarrhea was defined as KSC ≥ 16 for 3 days.

**Table 2:** King’s Stool Chart

Fecal consistency	Fecal weight		
	< 100 g	100–200g	> 200 g
Hard and formed	1	2	3
Soft and formed	2	3	4
Loose and unformed	4	6	8
Liquid	8	10	12

### Follow-up

For 5 years after surgery, CT was performed every 6 months, and EGD was performed yearly. Recurrence-free survival (RFS) was calculated from the day of surgery to the day of esophageal cancer or EGJC recurrence. Overall survival (OS) was calculated from the day of surgery to the day of death. Patients were followed up until death, five years after esophagectomy, or the study termination (December 31, 2022). Patients who were alive at the study termination, interrupted follow-up, and died due to an illness unrelated to their primary disease were recognized as censored.

### Statistical analysis

All statistical analyses were performed using IBM SPSS Statistics version 26 for Windows (IBM Corp., Armonk, NY, USA). Medians and ranges were calculated, and differences were identified using the Mann–Whitney *U* test. Differences between categorical variables were identified using the Chi-squared or Fisher’s exact test. Repeated measures analysis of variance was used to analyze the association between preoperative and postoperative nutritional status. Survival curves were generated using the Kaplan–Meier survival method and the log-rank test. Odds ratios (OR) and hazard ratios (HR) were calculated. Univariate and multivariate analyses were performed using logistic regression analysis for nominal variables and Cox proportional hazards regression models for survival analysis. Univariate and multivariate analyses of clinicopathologic factors that may be risk factors for diarrhea were performed using logistic regression analysis. The clinicopathological factors previously reported to be associated with prognosis, such as age, NAC, transthoracic approach, postoperative complications (pneumonia and AL), pathological stage, PNI, CRP, NLR, and diarrhea during EF, were evaluated by univariate and multivariate analysis using the Cox proportional hazards model. The threshold for significance was set at *p* < 0.05.

## Results

### Demographics and perioperative outcomes

Diarrhea occurred in 46 (30.3%) patients during EF, and patients were divided into a non-diarrhea (Group N, n = 106) and diarrhea group (Group D, n = 46) (Fig. 1). The clinicopathological features of each group are shown in Tables 3 and 4. This study defined elderly patients using a cut-off value of 70 years according to a previous study [21]. There was no significant difference in age, sex, histology, tumor location, clinical stage, or rate of NAC administration. Patients with a higher dysphagia score (≥ 1) were significantly found in Group D than in Group N (45.7% vs. 19.8%, *p* = 0.002) (Table 3). There were 19 (12.5%) patients who had reduced or stopped EF. Eleven (7.2%) patients had abdominal fullness and 8 (5.3%) patients had chylothorax. One of them was accompanied by abdominal fullness, chylothorax, and anorexia. However, all patients resumed EF after these symptoms improved. The morbidity of postoperative complications was similar between the two groups. In most patients, the oral diet was started on postoperative day 10 (median, range 7–35 days). The median duration from surgery to diarrhea onset was 10 days (range, 4–24 days), and it took several days after starting diarrhea management to achieve stool control (median 4 days, range 0–28 days). The duration of postoperative hospital stay was significantly longer in Group D compared to Group N (median 29 vs. 24 days, *p* = 0.011) (Table 4). Stool cultures were collected from 39 patients (28 in Group D and 11 in Group N), and revealed that *Enterococcus spp.* and *Escherichia coli* were frequently cultured in both groups. Furthermore, *Clostridioides difficile* and *Klebsiella oxytoca,* known to cause antibiotics-associated colitis, were found in a few patients in Group D (Online Resource 2).

**Table 3:** Preoperative features between Groups N and D

	Overall n = 152	Group N n = 106	Group D n = 46	p value
Age, years†	69.0 (32.0–85.0)	69.0 (32.0–85.0)	68.5 (46.0–85.0)	0.505
Sex (%)				
Male	132 (86.8%)	93 (87.7%)	39 (84.8%)	0.610
Female	20 (13.2%)	13 (12.3%)	7 (15.2%)	
Histology (%)				
Squamous cell carcinoma	125 (82.2%)	84 (79.2%)	41 (89.1%)	0.171
Adenocarcinoma	27 (17.8%)	22 (20.8%)	5 (10.9%)	
Location of tumor (%)				
Ut	24 (15.8%)	15 (14.2%)	9 (19.6%)	0.713
Mt	55 (36.2%)	37 (34.9%)	18 (39.1%)	
Lt	50 (32.9%)	37 (34.9%)	13 (28.3%)	
Ae	23 (15.1%)	17 (16.0%)	6 (13.0%)	
Clinical diagnosis^*^				
cT (%)				
cT1	59 (38.8%)	45 (42.5%)	14 (30.4%)	0.445
cT2	17 (11.2%)	12 (11.3%)	5 (10.9%)	
cT3	71 (46.7%)	45 (42.5%)	26 (56.5%)	
cT4	5 (3.3%)	4 (3.8%)	1 (2.2%)	
cN (%)				
cN0	74 (48.7%)	54 (50.9%)	20 (43.5%)	0.555
cN1	51 (33.6%)	36 (34.0%)	15 (32.6%)	
cN2	20 (13.2%)	12 (11.3%)	8 (17.4%)	
cN3	7 (4.6%)	4 (3.8%)	3 (6.5%)	
cStage (%)				
cStage I	56 (36.8%)	42 (39.6%)	14 (30.4%)	0.742
cStage II	28 (18.4%)	19 (17.9%)	9 (19.6%)	
cStage III	56 (36.8%)	37 (34.9%)	19 (41.3%)	
cStage IV	12 (7.9%)	8 (7.5%)	4 (8.7%)	
Neoadjuvant chemotherapy (%)				
−	84 (55.3%)	64 (60.4%)	20 (43.5%)	0.075
+	68 (44.7%)	42 (39.6%)	26 (56.5%)	
Dysphagia score, ≥ 1 (%)	42 (27.6%)	21 (19.8%)	21 (45.7%)	0.002
Preoperative body weight, kg†	57.9 (38.1–80.0)	59.1 (38.1–80.0)	53.6 (39.4–75.8)	0.010
Preoperative BMI, kg/m^2^†	21.5 (14.2–28.2)	21.8 (16.5–28.2)	20.5 (14.2–27.7)	0.031
Preoperative serum total protein, g/dL†	6.7 (5.6–7.9)	6.7 (5.8–7.9)	6.7 (5.6–7.8)	0.953
Preoperative serum albumin, g/dL†	4.1 (2.6–4.8)	4.1 (2.6–4.8)	4.1 (2.9–4.7)	0.577
Preoperative serum cholinesterase, U/L†	273.0 (90.0–532.0)	276.5 (97.0–442.0)	262.0 (90.0–532.0)	0.360
Preoperative serum CRP, mg/dL†	0.12 (0.02–5.25)	0.10 (0.02–5.25)	0.14 (0.02–3.90)	0.274
Preoperative PNI†	48.0 (31.4–58.1)	48.6 (31.4–58.1)	47.4 (37.1–55.2)	0.234
Preoperative NLR†	2.62 (0.67–14.67)	2.60 (0.67–14.67)	2.65 (0.77–10.17)	0.745

^†^Values are presented as median (range)

^*^Union for International Cancer Control TNM classification of malignant tumors 8th edition

*Ut* upper thoracic esophagus, *Mt* middle thoracic esophagus, *Lt* lower thoracic esophagus, *Ae* abdominal esophagus, *BMI* body mass index, *CRP* C-reactive protein, *PNI* prognostic nutritional index, *NLR* neutrophil lymphocyte ratio

**Table 4:** Surgical and pathological features between Groups N and D

	Overall n = 152	Group N n = 106	Group D n = 46	p value
Transthoracic approach (%)				
Thoracotomy	36 (23.7%)	21 (19.8%)	15 (32.6%)	0.100
MIE	116 (76.3%)	85 (80.2%)	31 (67.4%)	
Abdominal approach (%)				
Laparotomy	45 (29.6%)	31 (29.2%)	14 (30.4%)	1.000
Laparoscopy	107 (70.4%)	75 (70.8%)	32 (69.6%)	
LN dissection (%)				
2-field	54 (35.5%)	37 (34.9%)	17 (37.0%)	0.855
3-field	98 (64.5%)	69 (65.1%)	29 (63.0%)	
Reconstruct route (%)				
Retrosternal route	16 (10.5%)	10 (9.4%)	6 (13.0%)	0.568
Posterior mediastinal route	136 (89.5%)	96 (90.6%)	40 (87.0%)	
Operation time, min†	536.0 (390.0–744.0)	536.5 (390.0–732.0)	535.0 (414.0–744.0)	0.933
Blood loss, ml†	185 (10–2275)	175 (10–2275)	190 (45–2170)	0.185
Complications, C-D grade, ≥ II (%)				
Anastomotic leakage	18 (11.8%)	10 (9.4%)	8 (17.4%)	0.179
Pneumonia	32 (21.1%)	18 (17.0%)	14 (30.4%)	0.083
Surgical site infection	14 (9.2%)	11 (10.4%)	3 (6.5%)	0.554
*Clostridioides difficile* infection	2 (1.3%)	0 (0.0%)	2 (4.3%)	0.090
Therapeutic antibiotics (%)	92 (60.5%)	61 (57.5%)	31 (67.4%)	0.283
Maximum KSC score†	16 (1–100)	10 (1–48)	40 (16–100)	< 0.001
Hospital stays, day†	25.5 (15–73)	24 (15–60)	29 (18–73)	0.011
Postoperative oral diet start, day†	10 (7–35)	10 (7–33)	10 (7–35)	0.693
Pathological diagnosis^*^				
pT (%)				
pT0	6 (3.9%)	4 (3.8%)	2 (4.3%)	0.547
pT1	67 (44.1%)	50 (47.2%)	17 (37.0%)	
pT2	20 (13.2%)	15 (14.2%)	5 (10.9%)	
pT3	55 (36.2%)	35 (33.0%)	20 (43.5%)	
pT4	4 (2.6%)	2 (1.9%)	2 (4.3%)	
pN (%)				
pN0	57 (37.5%)	43 (40.6%)	14 (30.4%)	0.133
pN1	47 (30.9%)	34 (32.1%)	13 (28.3%)	
pN2	27 (17.8%)	19 (17.9%)	8 (17.4%)	
pN3	21 (13.8%)	10 (9.4%)	11 (23.9%)	
pStage (%)				
pStage 0	5 (3.3%)	4 (3.8%)	1 (2.2%)	0.327
pStage I	41 (27.0%)	30 (28.3%)	11 (23.9%)	
pStage II	39 (25.7%)	30 (28.3%)	9 (19.6%)	
pStage III	44 (28.9%)	30 (28.3%)	14 (30.4%)	
pStage IV	23 (15.1%)	12 (11.3%)	11 (23.9%)	
Initial recurrent site (%)**				
Local	7 (4.6%)	3 (2.9%)	4 (8.7%)	0.201
Regional LN	34 (22.4%)	16 (15.2%)	18 (39.1%)	0.003
Distant organ	31 (20.4%)	15 (14.3%)	16 (34.8%)	0.008
Death unrelated to primary disease (%)	6 (3.9%)	5 (4.7%)	1 (2.2%)	0.668

^†^ Values are presented as median (range)

^*^Union for International Cancer Control TNM classification of malignant tumors 8th edition

^*^^*^Multiple sites of recurrence existed in some patients

*MIE* minimal invasive esophagectomy, *LN* lymph node, *C-D* Clavien-Dindo, *KSC* King’s Stool Chart

### Loss of postoperative nutritional status due to diarrhea

**Fig. 2:**
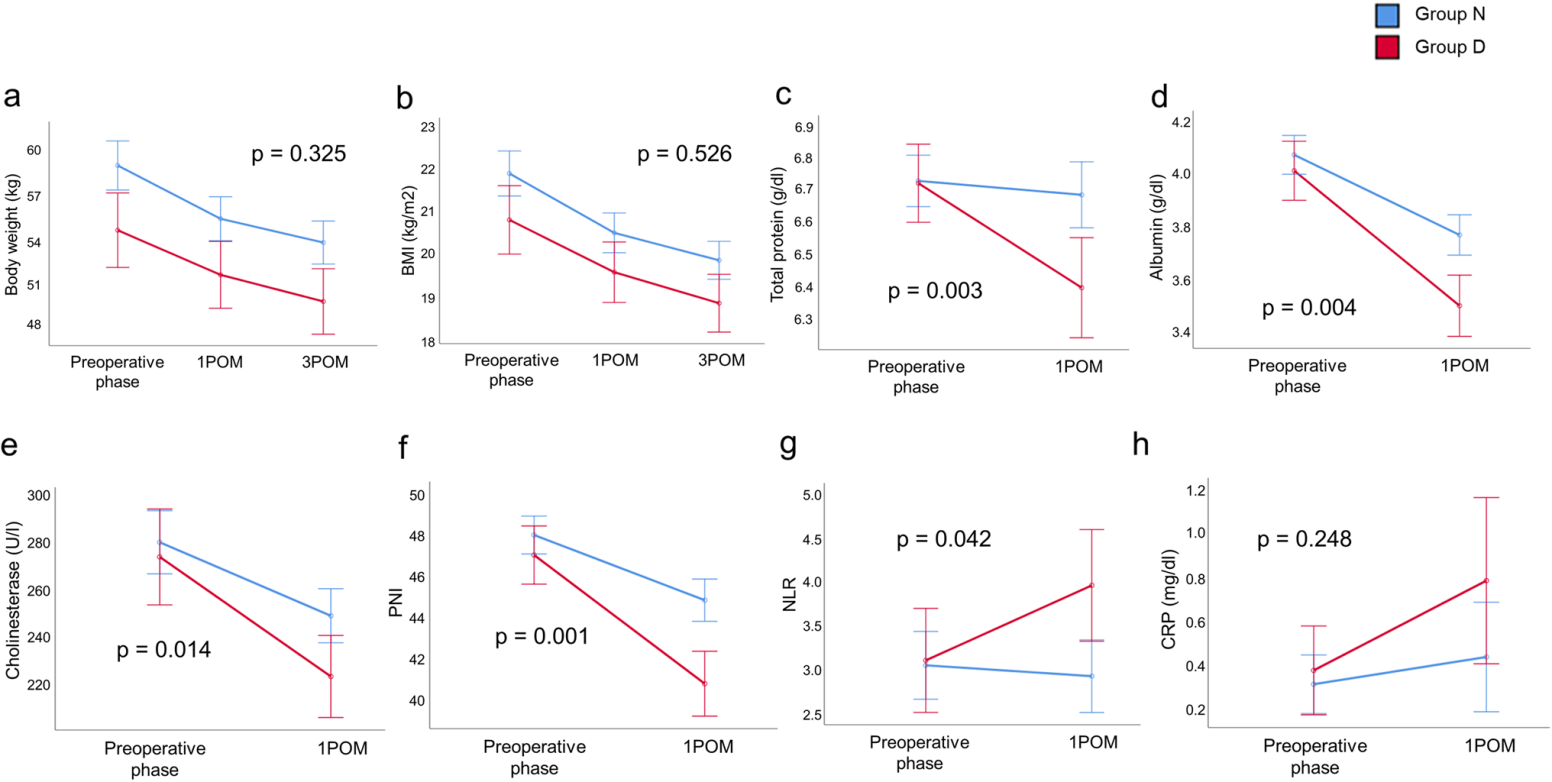
Changes in the nutritional status before and after esophagectomy. **a** Body weight. **b** Body mass index (BMI). **c** Serum total protein. **d** Serum albumin. **e** Serum cholinesterase. **f** Prognostic nutritional index (PNI). **g** Neutrophil-to-lymphocyte ratio (NLR). **h** C-reactive protein

The impact of diarrhea on postoperative nutritional status was investigated (Fig. 2). There was no significant difference in BW (*p* = 0.325) and BMI (*p* = 0.526) decline between the two groups before and at 1 and 3 months after surgery (Fig. 2a, b). However, compared to Group N, postoperative serum total protein (*p* = 0.003), serum albumin (*p* = 0.004), serum cholinesterase (*p* = 0.014), and PNI (*p* = 0.001) were significantly decreased in Group D (Fig. 2c–f). In addition, NLR values in Group N decreased from the preoperative phase to 1 month after surgery, whereas NLR values in Group D increased during the same period (*p* = 0.042) (Fig. 2g). There were no significant differences in serum CRP levels between the two groups (p = 0.248) (Fig. 2g).

Next, the patients were divided into two subgroups: those with or without postoperative infectious complications (AL, pneumonia, and SSI). In the non-infectious complication group, PNI (45.1 vs. 41.0, *p* = 0.009) and CRP (0.18 mg/dl vs. 0.34 mg/dl, *p* = 0.001) levels at 1 month after surgery were significantly better than those in the infectious complication group. However, there were no significant differences in NLR values between the two groups (2.81 in the non-infectious complication group vs. 2.90 in the infectious complication group, *p* = 0.360) (Online Resource 3). Additionally, multivariate analysis identified that laparotomy and diarrhea during EF were relevant factors for postoperative high NLR values (Online Resource 4).

### Risk factors for diarrhea during EF

Variables in Table 5 indicate potentially significant clinicopathological factors affecting diarrhea during EF. In multivariate analysis, a higher dysphagia score (≥ 1) (OR 3.781; *p* = 0.009; 95% confidence interval [CI] 1.398–10.227), NAC (OR 2.976; *p* = 0.040; 95% CI 1.050–8.436), and AL (OR 4.368; *p* = 0.022; 95% CI 1.232–15.488) were independent relevant factors for diarrhea during EF (Table 5). However, no correlation was observed between therapeutic antibiotic use and diarrhea (OR 1.349; *p* = 0.547; 95% CI 0.532–3.665).

**Table 5:** Independent relevant factors of clinicopathological features on diarrhea during EF

	Univariate analysis			Multivariate analysis		
	OR	P	95% CI	OR	P	95% CI
Age, ≥ 70	0.906	0.780	0.453–1.813	0.776	0.547	0.340–1.771
Dysphagia score, ≥ 1	3.400	0.001	1.604–7.208	3.781	0.009	1.398–10.227
Clinical stage, ≥ III	1.356	0.391	0.677–2.715	0.357	0.092	0.108–1.181
Neoadjuvant chemotherapy, +	1.981	0.056	0.983–3.993	2.976	0.040	1.050–8.436
Preoperative body weight, < median (57.9 kg)	1.880	0.079	0.929–3.804	2.157	0.082	0.908–5.125
Preoperative PNI, < median (48.0)	1.400	0.347	0.695–2.822	1.077	0.869	0.447–2.594
Preoperative NLR, ≥ median (2.62)	1.086	0.817	0.540–2.181	1.111	0.819	0.451–2.737
Preoperative CRP, positive	1.771	0.156	0.804–3.898	1.397	0.497	0.532–3.665
Transthoracic approach, Thoracotomy	1.959	0.091	0.898–4.272	2.222	0.084	0.900–5.488
Abdominal approach, Laparotomy	1.058	0.883	0.498–2.251	1.087	0.867	0.409–2.887
Postoperative pneumonia, +	2.139	0.065	0.954–4.794	2.593	0.058	0.969–6.937
Anastomotic leakage, +	2.021	0.169	0.741–5.509	4.368	0.022	1.232–15.488
Surgical site infection, +	0.603	0.454	0.160–2.270	0.222	0.089	0.039–1.255
Therapeutic antibiotics, +	1.525	0.255	0.737–3.154	1.349	0.547	0.532–3.665

*OR* odds ratio, *CI* confidence interval, *CRP* C-reactive protein, *PNI* prognostic nutritional index, *NLR* neutrophil-to-lymphocyte ratio, *CRP* C-reactive protein

### Survival analysis

The median follow-up period was 27.5 months (range 1.9–66.3 months); 52 (34.2%) patients experienced recurrence. Patients in Group D had significantly higher rates of regional LN and distant organ recurrence than those in Group N (regional LN recurrence: 39.1% vs. 15.2%, *p* = 0.003; distant organ recurrence: 34.8% vs. 14.3%, *p* = 0.008) (Table 4). The rate of patients dying from illnesses unrelated to primary disease was similar in both groups (Group D 2.2% vs. Group N 4.7%, *p* = 0.668).

**Fig. 3:**
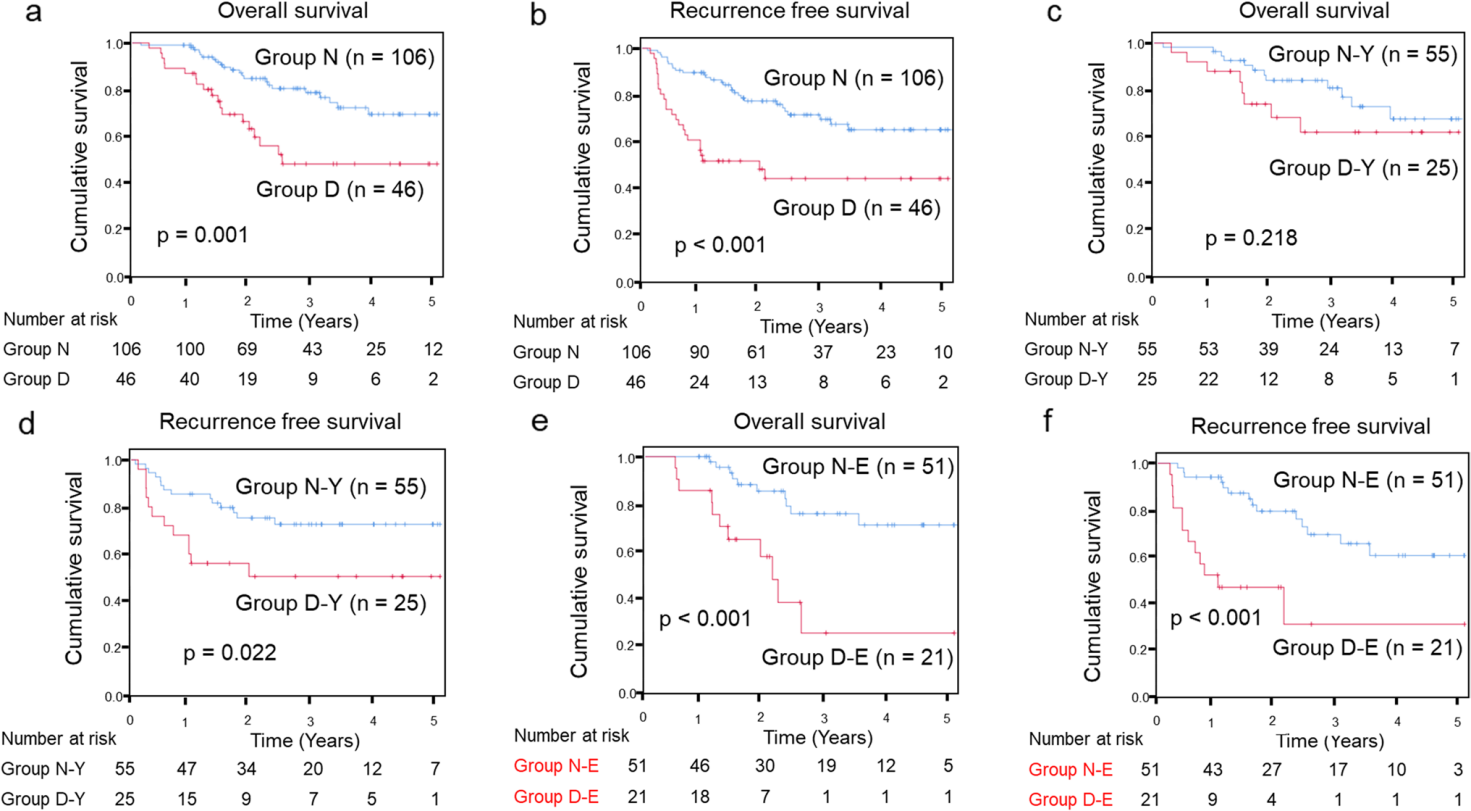
Kaplan–Meier analysis based on the incidence of diarrhea during enteral feedings. **a** Comparison of overall survival between Groups N and D. **b** Comparison of recurrence-free survival between Groups N and D.** c** Comparison of overall survival between the younger groups (Groups N-Y and D-Y). **d** Comparison of recurrence-free survival between the younger groups (Groups N-Y and D-Y). **e** Comparison of overall survival between the elderly groups (Groups N-E and D-E). **f** Comparison of recurrence-free survival between the elderly groups (Groups N-E and D-E)

Patients in Group D had significantly worse OS and RFS than those in Group N (median survival time (MST) OS: 21.9 vs. 30.6 months, *p* = 0.001; RFS: 12.4 vs. 27.7 months, *p* < 0.001) (Fig. 3a, b). In addition, an advanced pathological stage (≥ III) (HR 4.172; *p* = 0.001; 95% CI 1.749–9.947) and diarrhea during EF (HR 2.174; *p* = 0.040; 95% CI 1.038–4.456) were independent predictors of poor OS in the multivariate analysis (Table 6).

**Table 6:** Independent factors of clinicopathological features on poor overall survival

	Univariate analysis			Multivariate analysis		
	HR	P	95% CI	HR	P	95% CI
All cases (n = 152)						
Age, ≥ 70	1.468	0.215	0.800–2.694	1.483	0.235	0.774–2.843
Neoadjuvant chemotherapy, +	3.059	0.001	1.607–5.824	1.464	0.331	0.679–3.157
Transthoracic approach, Thoracotomy	1.182	0.635	0.593–2.354	1.009	0.982	0.485–2.098
Postoperative pneumonia, +	1.687	0.138	0.845–3.371	1.808	0.146	0.813–4.020
Anastomotic leakage, +	1.169	0.724	0.491–2.779	1.381	0.497	0.543–3.512
Diarrhea during EF	2.657	0.002	1.433–4.925	2.174	0.040	1.038–4.456
Pathological stage, ≥ III	5.235	< 0.001	2.478–11.057	4.172	0.001	1.749–9.947
POM1 PNI, < median (43.4)	1.037	0.907	0.565–1.903	0.655	0.269	0.411–1.842
POM1 NLR, < median (2.85)	1.155	0.742	0.490–2.723	0.870	0.716	0.411–1.842
POM1 CRP, positive	1.232	0.503	0.669–2.271	0.887	0.742	0.435–1.808
Younger patients (n = 80)						
Neoadjuvant chemotherapy, +	3.332	0.014	1.277–8.692	1.358	0.614	0.413–4.459
Transthoracic approach, Thoracotomy	1.795	0.231	0.689–4.680	1.195	0.749	0.400–3.572
Postoperative pneumonia, +	1.412	0.505	0.512–3.891	2.541	0.148	0.719–8.980
Anastomotic leakage, +	2.043	0.202	0.681–6.123	2.721	0.147	0.704–10.513
Diarrhea during EF	1.743	0.224	0.711–4.270	0.945	0.925	0.293–3.054
Pathological stage, ≥ III	4.656	0.003	1.689–12.836	4.732	0.017	1.323–16.929
POM1 PNI, < median (43.4)	1.212	0.671	0.499–2.939	0.323	0.056	0.101–1.208
POM1 NLR, < median (2.85)	1.529	0.352	0.625–3.744	1.851	0.252	0.646–5.300
POM1 CRP, positive	1.092	0.851	0.436–2.738	0.892	0.826	0.322–2.469
Elderly patients (n = 72)						
Neoadjuvant chemotherapy, +	2.766	0.024	1.145–6.679	1.076	0.903	0.334–3.467
Transthoracic approach, Thoracotomy	0.617	0.385	0.207–1.835	0.719	0.613	0.200–2.586
Postoperative pneumonia, +	2.125	0.122	0.818–5.519	2.375	0.210	0.614–9.185
Anastomotic leakage, +	0.554	0.428	0.128–2.386	0.802	0.783	0.167–3.852
Diarrhea during EF	4.369	0.001	1.814–10.526	3.717	0.040	1.059–13.042
Pathological stage, ≥ III	5.590	0.002	1.834–17.035	5.698	0.016	1.391–23.335
POM1 PNI, < median (43.4)	0.883	0.782	0.367–2.123	1.245	0.775	0.315–4.916
POM1 NLR, < median (2.85)	1.155	0.742	0.490–2.723	0.392	0.163	0.105–1.461
POM1 CRP, positive	1.359	0.484	0.576–3.205	0.818	0.754	0.233–2.875

*HR* hazard ratio, *CI* confidence interval, *EF* enteral feeding, *POM* post-operative month, *PNI* prognostic nutritional index, *NLR* neutrophil-to-lymphocyte ratio, *CRP* C-reactive protein

Next, the survival impact of the postoperative nutritional and immunological status (CRP, PNI, and NLR) was investigated. Although there were no significant differences, regarding CRP levels and NLR values, patients in the high group tended to have worse RFS than those in the low group (MST, CRP: 22.5 months in the high group vs. 23.3 months in the low group, *p* = 0.124; NLR: 22.4 months in the high group vs. 23.3 months in the low group, *p* = 0.198) (Online Resource 5).

### Survival impact of diarrhea on the elderly

The effect of diarrhea on prognosis was then evaluated depending on the age. The median age of the enrolled patients was 69 years, and the patients were divided into four subgroups: younger (< 70 years) non-diarrhea group (Group N-Y, n = 55), elderly (≥ 70 years) non-diarrhea group (Group N-E, n = 51), younger diarrhea group (Group D-Y, n = 25), and elderly diarrhea group (Group D-E, n = 21) (Fig. 1). Patients in Group D-Y had significantly worse RFS than in Group N-Y (MST: 15.7 vs. 29.3 months, *p* = 0.022); nevertheless, there was no significant difference in OS between Groups D-Y and N-Y (MST: 24.1 vs. 33.6 months, *p* = 0.218) (Fig. 3c, d). However, patients in Group D-E had significantly worse OS and RFS than those in Group N-E (MST OS: 17.8 vs. 27.9 months, *p* < 0.001; RFS: 11.9 vs. 26.9 months, *p* < 0.001) (Fig. 3e, f). Finally, clinicopathological variables potentially affecting poor OS were investigated (Table 6). In the younger group, the multivariate analysis identified an advanced pathological stage (≥ III) (HR 4.732; *p* = 0.017; 95% CI 1.323–16.929) as an independent predictive factor for poor OS (Table 6). Diarrhea during EF was not associated with poor OS. However, in the elderly group, the multivariate analysis identified that an advanced pathological stage (≥ III) (HR 5.698; *p* = 0.016; 95% CI 1.391–23.335) and diarrhea during EF (HR 3.717; *p* = 0.040; 95% CI 1.059–13.042) were independent predictive factors for poor OS (Table 6).

## Discussion

This study demonstrated that patients with diarrhea during EF after esophagectomy had significantly worse OS and RFS than those without diarrhea. Multivariate analysis revealed that diarrhea during EF was an independent prognostic factor for poor OS after esophagectomy. Furthermore, the nutritional status in Group D significantly decreased compared with that in Group N. To the best of our knowledge, this is the first report to reveal the survival impact of diarrhea during EF after esophagectomy.

Postoperative malnutrition has been reported to result in immune function deficiency and chemotherapy intolerance [5, 22]. Additionally, NLR increased only in Group D from the preoperative phase to 1 month after esophagectomy. Postoperative NLR values were similar between the postoperative infectious and non-infectious complication groups. Additionally, multivariate analysis identified that laparotomy and diarrhea during EF were relevant factors for high postoperative NLR values. These results suggested that postoperative neutrophil production was not associated with postoperative infectious complications but with diarrhea during EF. Furthermore, compared with that in the low NLR group, the Kaplan–Meier curve for RFS showed a decline in the high NLR group. Diarrhea during EF may induce inflammation, increasing neutrophil production and NLR elevations. Neutrophils induce chemokines and cytokine production, which enhances tumor growth, invasion, and angiogenesis [23]. These results suggest that diarrhea during EF can cause postoperative inflammation and malnutrition, resulting in decreased tumor immunity and a poor prognosis.

The stratified analysis revealed that RFS in the diarrhea group was significantly worse than that in the non-diarrhea group both in the younger and elderly groups. In addition, patients in the diarrhea group had significantly worse OS than those in the non-diarrhea group in the elderly group. Furthermore, the multivariate analysis revealed diarrhea during EF as an independent risk factor for poor OS in the elderly. Elderly patients usually have reduced satiety and poor digestive function, resulting in long-term reduced oral intake and malnutrition [11, 24]. Additionally, physiological function, especially organ reserve, declines with age, and comorbidities and frailty become increasingly common as people age. The decline in organ reserve becomes apparent only after stresses such as surgery or chemotherapy [25]. These findings suggest that diarrhea may have devastating effects on postoperative quality of life, physical strength, and prognosis, especially in the elderly.

Diarrhea during EF was associated with a higher dysphagia score. There is evidence that loss of oral intake can cause digestive and absorptive capacity deterioration due to intestinal villus atrophy [24, 26]. The esophageal cancer mass frequently obstructs the passage of solids before surgery, which causes atrophy of the intestinal villus and increases the risk of postoperative diarrhea.

Diarrhea can potentially disrupt the microbiota. By modulating the immune function of the host, intestinal bacteria can improve the immune system’s defense against cancer [27]. Intestinal microbiota disturbance can lead to the development of several pathologies, including malnourishment and chronic inflammatory disorders, such as inflammatory bowel disease, which have a significant impact on colorectal cancer pathogenesis [27, 28]. These findings suggest that diarrhea during EF may be a risk factor for intestinal microbiota distribution, malnutrition, and immunological disorders, contributing to a poor prognosis after esophagectomy.

This study found that there was no relationship between therapeutic antibiotic use and diarrhea. Antibiotics can negatively affect the gut microbiota, causing pathogenic bacteria, including *Clostridioides difficile*, to proliferate [29, 30]. However, few patients in this study had pathogenic bacteria that caused antibiotics-associated colitis, and resident bacteria in the intestinal tract were cultured in many patients. These findings suggest that diarrhea during EF was predominantly osmotic.

Diarrhea can be managed by slowing EF infusion or changing enteral nutritional supplements [31]. However, the frequency of diarrhea is often not accurately assessed and may be overlooked. Furthermore, an improvement in stool condition required several days from the start of diarrhea management in this study. Therefore, we formed a multidisciplinary support team and shared information on the nutritional status, dietary intake, selection of EF agents, and diarrhea at the conference [6, 18]. Regarding the administration of EF, it is important not only to adjust the rate of EF and agents according to abdominal symptoms but also to manage diarrhea during NAC. It is essential to communicate information about abdominal symptoms during EF with the multidisciplinary team and to administer prophylactic intestinal regimens.

This study has some limitations. First, this was a single-institution retrospective study. However, this study reviewed consecutive patients, which reduced selection bias. Second, there was some variability because the diagnosis of diarrhea was based on the subjective records of the nurse. In practice, there was a delay from detection to therapeutic intervention in some cases. To reduce bias among nurses, diarrhea was defined as the occurrence of continuous liquid stool for 3 days in this study. Finally, some patients were under treatment for recurrence at the conclusion of this study. Therefore, the effect of diarrhea on chemotherapy tolerance and the response rate for recurrence remains unclear. Continuous follow-ups and a multi-institutional prospective study should validate the current findings in the future.

In conclusion, diarrhea during EF can induce postoperative malnutrition, which leads to a poor prognosis after esophagectomy, especially in the elderly. Therefore, precise observation of the patient’s condition after esophagectomy may be essential in preventing EF-related diarrhea and a multidisciplinary support team may play an important role.

### Supplementary Information

Below is the link to the electronic supplementary material.

Supplementary file1 (PDF 120 KB)

Supplementary file2 (PDF 159 KB)

Supplementary file3 (PDF 171 KB)

Supplementary file4 (PDF 159 KB)

Supplementary file5 (PDF 288 KB)

## Data Availability

The datasets generated and/or analyzed during the current study are available from the corresponding author upon reasonable request.
