# Supplementary material for: Clinical impact of diarrhea during enteral feeding after esophagectomy
Source: Int J Clin Oncol. 2023 Nov 23;29(1):36–46. doi: 10.1007/s10147-023-02428-5 (PMC10764458; doi:10.1007/s10147-023-02428-5)
Supplement: Supplementary file 1 — Supplementary file1 (PDF 120 KB) [file 10147_2023_2428_MOESM1_ESM.pdf]

Online Resource 1 Feeding strategy

Postoperative day (POD)	POD 0	POD 1	POD 2	POD 3	POD 4－6	POD 7－8
Element diet	240 kcal	480 kcal	720 kcal	960 kcal	1200 kcal	1000 kcal
Fat-containing diet	None	None	None	None	None	200 kcal
Oral diet	None	None	None	None	None	None

Postoperative day (POD)	POD 9	POD 10	POD 11	POD 12	POD 13－Discharge
Element diet	900 kcal	900 kcal	300 kcal	300 kcal	None
Fat-containing diet	400 kcal	400 kcal	600 kcal	600 kcal	600 kcal
Oral diet	None	Started	Started	Started	Started

Clinical impact of diarrhea during enteral feeding after esophagectomy

Ryoma Haneda, MD[1], Yoshihiro Hiramatsu, MD, Ph.D[1,2], Sanshiro Kawata, MD, Ph.D[1], Wataru Soneda, MD[1], Eisuke Booka, MD, Ph.D[1], Tomohiro Murakami, MD, Ph.D[1], Tomohiro Matsumoto, MD, Ph.D[1], Yoshifumi Morita, MD, Ph.D[1], Hirotoshi Kikuchi, MD, Ph.D[1], and Hiroya Takeuchi, MD, Ph.D[1]

1. Department of Surgery, Hamamatsu University School of Medicine, Hamamatsu, Shizuoka, Japan

2. Department of Perioperative Functioning Care and Support, Hamamatsu University School of Medicine, Hamamatsu, Shizuoka, Japan

Corresponding author: Yoshihiro Hiramatsu, MD, Ph.D.

Department of Perioperative Functioning Care and Support, Hamamatsu University School of Medicine

1-20-1 Handayama, Higashi-ku, Hamamatsu, Shizuoka 431-3192, Japan

E-mail: hiramatu@hama-med.ac.jp

Phone: +81-53-435-2427; Fax: +81-53-435-2423
