# Supplementary material for: Clinical impact of diarrhea during enteral feeding after esophagectomy
Source: Int J Clin Oncol. 2023 Nov 23;29(1):36–46. doi: 10.1007/s10147-023-02428-5 (PMC10764458; doi:10.1007/s10147-023-02428-5)
Supplement: Supplementary file 2 — Supplementary file2 (PDF 159 KB) [file 10147_2023_2428_MOESM2_ESM.pdf]

Online Resource 2 Stool culture

Group D<br>n = 28		Group N<br>n = 11	
Bacterium	Number (%)	Bacterium	Number (%)
Enterococcus	15 (53.4%)	*Enterococcus*	6 (54.5%)
Escherichia coli	9 (32.1%)	*Escherichia coli*	4 (36.4%)
Klebsiella pneumoniae	7 (25.0%)	*Culture negative*	2 (18.2%)
Candida	5 (17.9%)	*Klebsiella pneumoniae*	1 (9.1%)
Pseudomonas	5 (17.9%)	*Candida*	1 (9.1%)
Citrobacter	2 (7.1%)	*Citrobacter*	1 (9.1%)
Klebsiella oxytoca	2 (7.1%)	*Serratia*	1 (9.1%)
Enterobacter cloacae	2 (7.1%)	*Aeromonas*	1 (9.1%)
Clostridium difficile	2 (7.1%)		
Stenotrophomonas maltophilia	2 (7.1%)		
Lactobacillus	1 (3.6%)		
Proteus	1 (3.6%)		
Serratia	1 (3.6%)		
Streptococcus	1 (3.6%)		
Acinetobacter	1 (3.6%)		
Raoultella ornithinolytica	1 (3.6%)		
Morganella morganii	1 (3.6%)		

Clinical impact of diarrhea during enteral feeding after esophagectomy

Ryoma Haneda, MD[1], Yoshihiro Hiramatsu, MD, Ph.D[1,2], Sanshiro Kawata, MD, Ph.D[1], Wataru Soneda, MD[1], Eisuke Booka, MD, Ph.D[1], Tomohiro Murakami, MD, Ph.D[1], Tomohiro Matsumoto, MD, Ph.D[1], Yoshifumi Morita, MD, Ph.D[1], Hirotoshi Kikuchi, MD, Ph.D[1], and Hiroya Takeuchi, MD, Ph.D[1]

1. Department of Surgery, Hamamatsu University School of Medicine, Hamamatsu, Shizuoka, Japan

2. Department of Perioperative Functioning Care and Support, Hamamatsu University School of Medicine, Hamamatsu, Shizuoka, Japan

Corresponding author: Yoshihiro Hiramatsu, MD, Ph.D.

Department of Perioperative Functioning Care and Support, Hamamatsu University School of Medicine

1-20-1 Handayama, Higashi-ku, Hamamatsu, Shizuoka 431-3192, Japan

E-mail: hiramatu@hama-med.ac.jp

Phone: +81-53-435-2427; Fax: +81-53-435-2423
