# Supplementary material for: Clinical impact of diarrhea during enteral feeding after esophagectomy
Source: Int J Clin Oncol. 2023 Nov 23;29(1):36–46. doi: 10.1007/s10147-023-02428-5 (PMC10764458; doi:10.1007/s10147-023-02428-5)
Supplement: Supplementary file 3 — Supplementary file3 (PDF 171 KB) [file 10147_2023_2428_MOESM3_ESM.pdf]

Online Resource 3 Association between postoperative infectious complications and clinical factor

	Non-infectious complication group n = 94	Infectious complication group n = 58	p value
Transthoracic approach (%)			0.240
Thoracotomy	19 (20.2%)	17 (29.3%)	
MIE	75 (79.8%)	41 (70.7%)	
Abdominal approach (%)			0.201
Laparotomy	24 (25.5%)	21 (36.2%)	
Laparoscopy	70 (74.5%)	37 (63.8%)	
Operation time, min†	537.5 (390.0－732.0)	535.0 (419.0－744.0)	0.254
Blood loss, ml†	161 (10－2275)	235 (35－2170)	0.024
Maximum KSC score†	16 (1－100)	16 (2－100)	0.125
POM1, CRP†	0.18 (0.03－14.32)	0.34 (0.07－2.58)	0.001
POM1 PNI†	45.1 (32.8－58.1)	41.0 (29.3－56.3)	0.009
POM1 NLR†	2.81 (0.71－19.79)	2.90 (0.56－8.03)	0.360

† Values are presented as median (range)

MIE, minimal invasive esophagectomy; *KSC*, King's Stool Chart; *POM*, post-operative month; *CRP*, C-reactive protein; *PNI*, prognostic nutritional index; *NLR*, neutrophil-to-lymphocyte ratio

Clinical impact of diarrhea during enteral feeding after esophagectomy

Ryoma Haneda, MD[1], Yoshihiro Hiramatsu, MD, Ph.D[1,2], Sanshiro Kawata, MD, Ph.D[1], Wataru Soneda, MD[1], Eisuke Booka, MD, Ph.D[1], Tomohiro Murakami, MD, Ph.D[1], Tomohiro Matsumoto, MD, Ph.D[1], Yoshifumi Morita, MD, Ph.D[1], Hirotoshi Kikuchi, MD, Ph.D[1], and Hiroya Takeuchi, MD, Ph.D[1]

1. Department of Surgery, Hamamatsu University School of Medicine, Hamamatsu, Shizuoka, Japan

2. Department of Perioperative Functioning Care and Support, Hamamatsu University School of Medicine, Hamamatsu, Shizuoka, Japan

Corresponding author: Yoshihiro Hiramatsu, MD, Ph.D.

Department of Perioperative Functioning Care and Support, Hamamatsu University School of Medicine

1-20-1 Handayama, Higashi-ku, Hamamatsu, Shizuoka 431-3192, Japan

E-mail: hiramatu@hama-med.ac.jp

Phone: +81-53-435-2427; Fax: +81-53-435-2423
