# Supplementary material for: Clinical impact of diarrhea during enteral feeding after esophagectomy
Source: Int J Clin Oncol. 2023 Nov 23;29(1):36–46. doi: 10.1007/s10147-023-02428-5 (PMC10764458; doi:10.1007/s10147-023-02428-5)
Supplement: Supplementary file 4 — Supplementary file4 (PDF 159 KB) [file 10147_2023_2428_MOESM4_ESM.pdf]

Online Resource 4 Independent relevant factors of postoperative high NLR

	Univariate analysis			Multivariate analysis		
	OR	P	95% CI	OR	P	95% CI
Age, ≥ 70	1.111	0.745	0.588－2.101	1.090	0.800	0.559－2.126
Transthoracic approach, Thoracotomy	0.864	0.703	0.409－1.828	0.820	0.626	0.368－1.825
Abdominal approach, Laparotomy	2.314	0.022	1.126－4.753	2.303	0.029	1.090－4.864
Postoperative infectious complications, +	1.250	0.504	0.649－2.409	1.004	0.991	0.498－2.025
Diarrhea during EF	2.446	0.015	1.193－5.015	2.577	0.014	1.213－5.472

NLR, neutrophil-to-lymphocyte ratio; *OR*, odds ratio; *CI*, confidence interval; *EF,* enteral feeding

Clinical impact of diarrhea during enteral feeding after esophagectomy

Ryoma Haneda, MD[1], Yoshihiro Hiramatsu, MD, Ph.D[1,2], Sanshiro Kawata, MD, Ph.D[1], Wataru Soneda, MD[1], Eisuke Booka, MD, Ph.D[1], Tomohiro Murakami, MD, Ph.D[1], Tomohiro Matsumoto, MD, Ph.D[1], Yoshifumi Morita, MD, Ph.D[1], Hirotoshi Kikuchi, MD, Ph.D[1], and Hiroya Takeuchi, MD, Ph.D[1]

1. Department of Surgery, Hamamatsu University School of Medicine, Hamamatsu, Shizuoka, Japan

2. Department of Perioperative Functioning Care and Support, Hamamatsu University School of Medicine, Hamamatsu, Shizuoka, Japan

Corresponding author: Yoshihiro Hiramatsu, MD, Ph.D.

Department of Perioperative Functioning Care and Support, Hamamatsu University School of Medicine

1-20-1 Handayama, Higashi-ku, Hamamatsu, Shizuoka 431-3192, Japan

E-mail: hiramatu@hama-med.ac.jp

Phone: +81-53-435-2427; Fax: +81-53-435-2423
