# Supplementary material for: Clinical impact of diarrhea during enteral feeding after esophagectomy
Source: Int J Clin Oncol. 2023 Nov 23;29(1):36–46. doi: 10.1007/s10147-023-02428-5 (PMC10764458; doi:10.1007/s10147-023-02428-5)
Supplement: Supplementary file 5 — Supplementary file5 (PDF 288 KB) [file 10147_2023_2428_MOESM5_ESM.pdf]

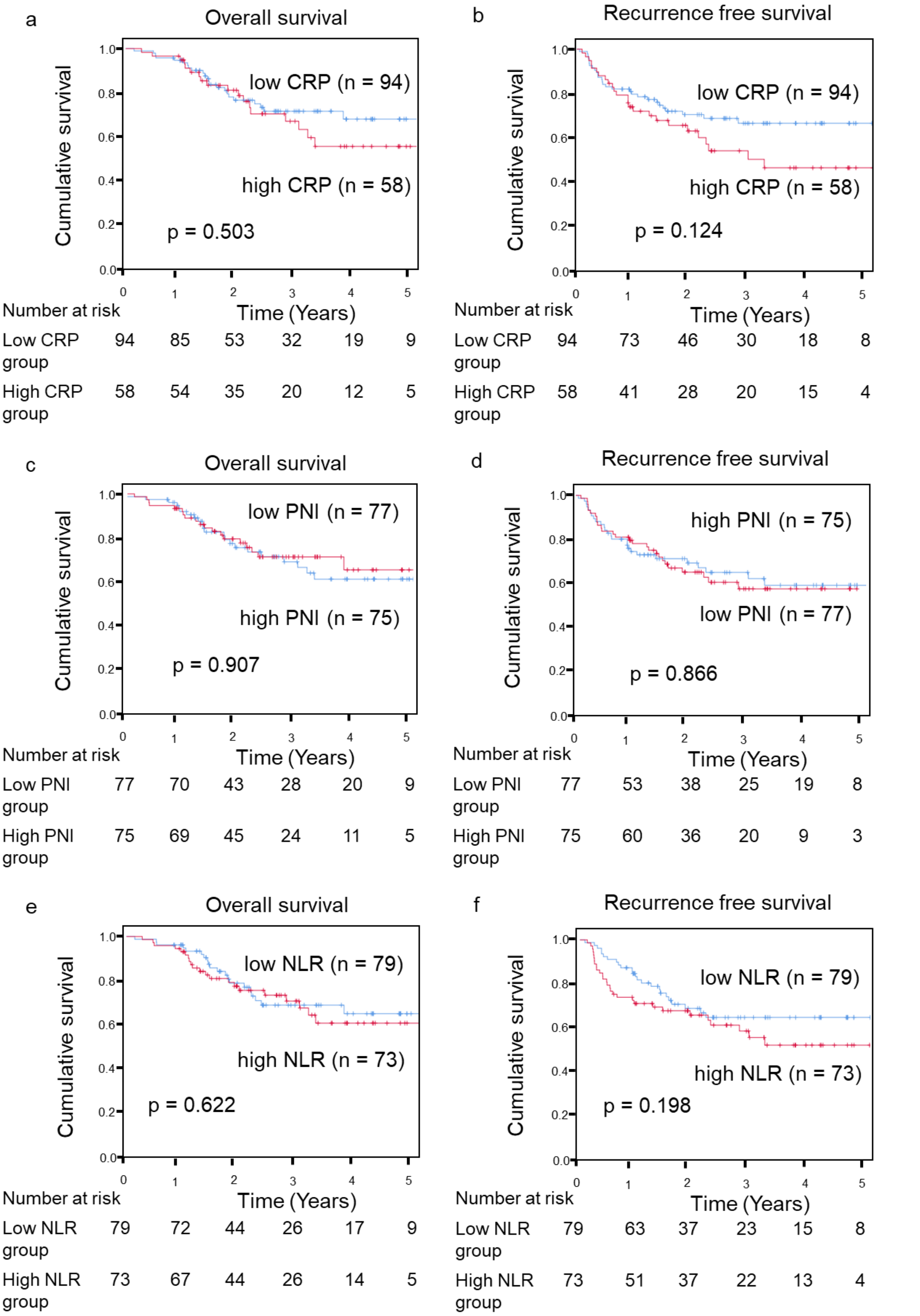

Online Resource 5. Kaplan–Meier analysis based on the postoperative nutritional and immunological status. **a**. Comparison of overall survival between low- and high-CRP

groups. **b**. Comparison of recurrence-free survival between low- and high-CRP groups. **c**. Comparison of overall survival between low- and high-PNI groups. **d**. Comparison of recurrence-free survival between low- and high-PNI groups. **e**. Comparison of overall survival between low- and high-NLR groups. **f**. Comparison of recurrence-free survival between low- and high-NLR groups.

Clinical impact of diarrhea during enteral feeding after esophagectomy

Ryoma Haneda, MD[1], Yoshihiro Hiramatsu, MD, Ph.D[1,2], Sanshiro Kawata, MD, Ph.D[1], Wataru Soneda, MD[1], Eisuke Booka, MD, Ph.D[1], Tomohiro Murakami, MD, Ph.D[1], Tomohiro Matsumoto, MD, Ph.D[1], Yoshifumi Morita, MD, Ph.D[1], Hirotoshi Kikuchi, MD, Ph.D[1], and Hiroya Takeuchi, MD, Ph.D[1]

1. Department of Surgery, Hamamatsu University School of Medicine, Hamamatsu, Shizuoka, Japan

2. Department of Perioperative Functioning Care and Support, Hamamatsu University School of Medicine, Hamamatsu, Shizuoka, Japan

Corresponding author: Yoshihiro Hiramatsu, MD, Ph.D.

Department of Perioperative Functioning Care and Support, Hamamatsu University School of Medicine

1-20-1 Handayama, Higashi-ku, Hamamatsu, Shizuoka 431-3192, Japan

E-mail: hiramatu@hama-med.ac.jp

Phone: +81-53-435-2427; Fax: +81-53-435-2423
